# Addressing the gap for racially diverse research involvement: The King's Model for minority ethnic research participant recruitment^☆^

**DOI:** 10.1016/j.puhip.2023.100426

**Published:** 2023-09-10

**Authors:** K. Ray Chaudhuri, A. Podlewska, Yue Hui Lau, C. Gonde, A. McIntosh, M.A. Qamar, S. O'Donoghue, K. Larcombe, M. Adeeko, A. Gupta, S. Bajwah, S. Lafond, O. Awogbemila, R. van Coller, A.M. Murtagh, J.E. Ocloo

**Affiliations:** a Institute of Psychiatry, Psychology & Neuroscience, King's College London, London, United Kingdom; b National Institute for Health and Care Research (NIHR), Applied Research Collaboration South London (NIHR ARC South London), King's College Hospital NHS Foundation Trust, London, United Kingdom; c King's College Hospital NHS Foundation Trust, London, United Kingdom; d Institute of Liver Studies, King's College Hospital, NHS Trust Foundation, London, United Kingdom; e Paediatric Respiratory Medicine, King's College Hospital, London, United Kingdom; f King's Health Partners, London, SE1 9RT, United Kingdom; g Cicely Saunders Institute, King's College London, London, United Kingdom; h Department of Neurology, Faculty of Health Sciences, University of Pretoria, South Africa

**Keywords:** Research participation, Ethnic minorities, Diversity, Inclusion

## Abstract

**Objectives:**

Ethnic minorities (EM) are still underrepresented in research recruitment. Despite wide literature outlining the barriers, enablers and recommendations for driving inclusion and diversity in research, there is still little evidence for successful diversity in research participation, which has a direct impact on the quality of care provided to ethnically diverse individuals. A new, comprehensive approach to recruitment strategies is therefore necessary.

**Study design:**

service improvement initiative.

**Methods:**

In the light of the Covid-19 pandemic and the key public health need to address the disparity in care provided to non-white populations, we used a novel, comprehensive approach (The King's Model) comprising of local and community actions to promote inclusive research recruitment. We then compared rates of diverse recruitment in studies where the novel approach, was applied to studies which had been closed to recruitment at the time of analysis and where ethnicity data was available.

**Results:**

Our results demonstrate that following the introduction of the King's Model for diverse recruitment, commercial interventional study diverse recruitment increased from 6.4% to 16.1%, and for non-commercial studies, from 30.2% to 41.0% and 59.2% in the selected studies.

**Conclusions:**

King's Model is potentially a useful tool in enhancing non-Caucasian recruitment to clinical research. Enriched by additional recommendations based on our experiences during the Covid-19 research recruitment drive, we propose the King's Model is used to support ethnically diverse research recruitment. Further evidence is needed to replicate our findings, although this preliminary evidence provides granular details necessary to address the key unmet need of validating clinical research outcomes in non-white populations.

## 1 Background

It is evident that research participation varies greatly among different ethnicities. This has a direct effect on healthcare experiences and the extent to which personalised medicine can be applied patients of ethnic minorities (EM).

Despite the work carried out over the years [2] and a wide literature existing concerning the need for inclusion of EM in research [3], recent report demonstrates that little effort has been made to promote diversity in research and in real terms, where diverse populations are available, research recruitment remains largely limited to white Caucasian, middle class, educated individuals [4]. In addition, a pre-pandemic systematic review investigating researcher reported strategies for non-white recruitment into clinical trials in the United Kingdom highlighted that limited strategies have been employed to increase recruitment among ethnic minorities [5]. Recently, the American Thoratic Society proposed a multilevel framework to engage minority populations in clinical research across pulmonary, critical care and sleep medicine [6], though further research is needed to explore its effectiveness and global applicability.

With the recent Covid-19 pandemic, and the rate of severe infections and mortality being higher among EM populations [7,8], ethnically diverse recruitment to research became the key need which had to be addressed [9]. Given the diverse population residing in South-East London and within the catchment area of King's College Hospital (KCH), we aimed to increase EM recruitment into medical research by applying a novel, holistic approach for promoting research, encompassing both local (in-hospital) and community actions focusing on creating open dialogues with the clinical teams and local populations to bridge the gap and address any barriers that may be preventing EM populations from taking part in clinical research.

This article outlines a novel, holistic approach to addressing the current gap in research recruitment diversity, applied in a real-life, people-based setting. We applied the herewith proposed model to research studies conducted at KCH, and the aims were to (i) observe an increased recruitment to Covid-19 studies among EM populations and (ii) explore the feasibility of implementation of the model to future research participation strategies. We propose that this approach, called the King's Model, may be useful in increasing clinical trial participation among EM populations.

## 2 Methods

### 2.1 Model development

**Fig. 1:**
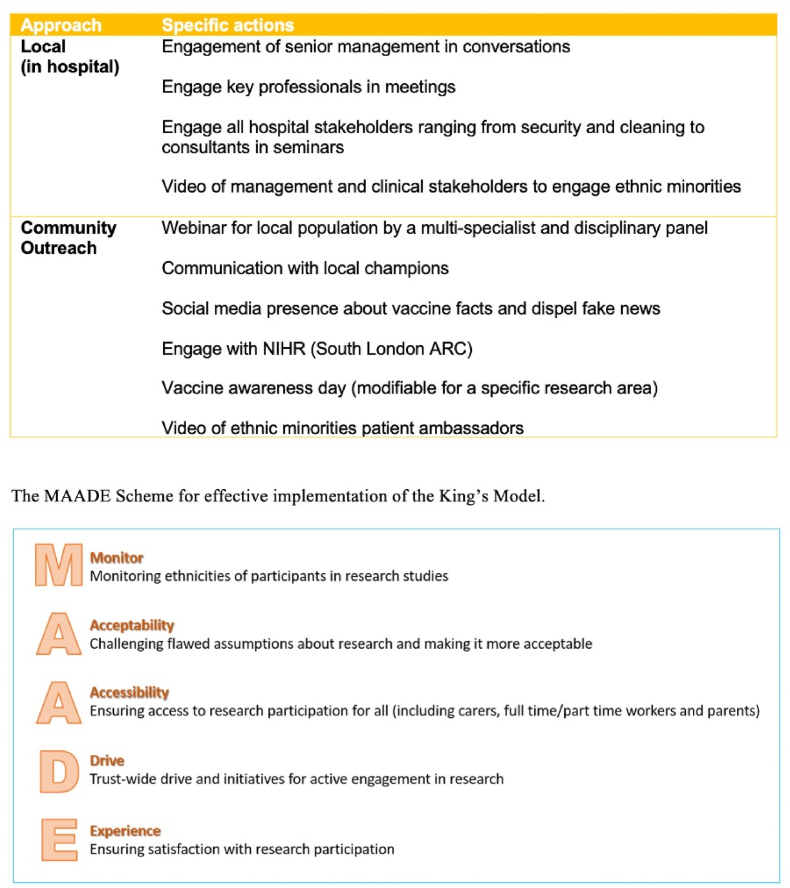
An outline of the King's Model for Research Participation Recruitment, outlining local and community steps to increase diverse research recruitment, along with the MAADE scheme, a set of considerations for long-term implementation.

The model was developed with the local PPI group and the Equality, Diversity and Inclusion strategy team at KCH, in a series of focus groups and with the aid of literature outlined above. It aims to propose a set of actions, both internal and external, which, when employed, can help increase EM participation in research locally. Summary figure of the Model items outlined in Fig. 1.

### 2.2 Model implementation

Where available, we inspected diversity in retrospective research recruitment to studies conducted at KCH NHS Foundation Trust within the period of 2017–2020, across a number of specialities including neurology, stroke, cardiology and palliative care.

Concurrently, we applied the strategies as outlined in Fig. 1 of the King's Model to research recruitment drive for three key Covid-19 studies, given their public health urgent need status. We implemented items from the proposed Model starting in September 2020 and analysed the data in September 2022. Per items outlined in Fig. 1, King's Model was applied in two dimensions: local (in hospital) action and a community outreach.

### 2.3 Local action

This focused around increasing research awareness, internally involving all hospital stakeholders in promoting research and addressing misconceptions that the public may have around research through:

• Creation of electronic resources imperative to driving diverse research participation within the hospital
• Attendance of meetings and seminars where we highlighted the issues to senior management to create a line of dialogue
• Attendance of smaller departmental meetings to educate staff on the current issues and hear their views
• Organisation of meetings focused on diverse recruitment strategies with key professionals with a track record of working on this issue

### 2.4 Community outreach

We targeted the local community groups including churches, schools and colleges and youth centres, to:

• Identify lay, local champions who can promote research
• Hold webinars for local population featuring a multi-disciplinary panel to increase the awareness and dispel any misconceptions
• Hold Vaccine Awareness Day to drive recruitment to Covid-19 vaccine trials, with key staff manning stalls open for public to visit to create opportunities to address any questions that the general population may have
• Disseminate a series of videos featuring ethnic minorities patient ambassadors and increase our social media presence, ensuring that the local reach is intensified and that the key hashtags and localisation services are utilised.

### 2.5 Assessing effectiveness

As the Model was first applied during the unprecedented time of the Covid-19 pandemic, we decided to focus on the high-profile Public Health England studies and target recruitment to those, given the urgent need for EM participants. The retrospective recruitment data was gathered on the basis of availability, where ethnicity data was accessible from the KCH study portfolio.

## 3 Results

Prior to initiation and implementation of the King's Model, we had performed a rough signposting of the current landscape of ethnic minority (EM) population participation in research trials within the Trust using existing research databases among different research delivery units, where data on race identification was available for use and disclosure. Robust data was available in selected research and when looking at commercial interventional studies, it was clear that within four research delivery units, representing 78 patients, while 93.6% were White Caucasian, only 6.4% were EM. Similarly, when considering non-commercial studies, from a total pool of 901 participants, recruitment of EM participants was higher at 30.2%, but still significantly lower when considering the local catchment area, where non-white individuals make up on average, 46.2% of the total population in Lambeth, Southwark and Croydon [10].

We selected out three high profile Public Health England studies taking place at KCH to utilise King's Model: PHOSP-COVID (IRAS: 285439, non-commercial observational), COVID-CNS (UKRI/MRC (MR/V03605X/1, non-commercial observational), and NOVAVAX (IRAS: 288793, commercial interventional). Following application of King's model, we found that the percentage of recruitment of EM participants to commercial trials increased to 16.1% (King's Model applied to the NOVAVAX trial) and in the targeted non-commercial studies, ME recruitment increased to 41.0% (PHOSP-COVID) and 59.2% (COVID-CNS). Due to a small sample of studies, the observed differences were not tested for statistical significance.

Recruitment data was collected from central records kept by the King's Research and Development department. A health inequalities oversight group provided advice when required.

## 4 Conclusions

This work outlines the premises of the King's Model for Minority Ethnic Research Participant Recruitment, based on the work undertaken at King's College Hospital since 2020, and builds on the proposals made globally, which often stem from detailed work with focus groups [1,6]. The current report presents real life, people-based strategies while outlining, in granular details, the necessary action points (the MAADE Scheme) when considering implementation of such Model to increase EM participation in clinical trials. It is therefore imperative that our proposal is rooted in clinical practice, which makes it unique compared to the other frameworks currently available.

The King's Model was initially used in the first stages of Covid-19 research projects, with PHOSP-COVID being the first targeted study. Given vaccination hesitancy among non-white populations, the model was also utilised with the Novavax interventional trial and at a later stage, with the observational COVID-CNS study. The implementation of the individual items outlined in the Model was conducted simultaneously to increase reach and ensure that the drive for diverse recruitment was in focus both locally and in the community. Consequently, our local recruitment represents higher numbers of EM participation, compared to studies where the Model was not utilised previously.

Broadly, the action, outlined as items in the Model (Fig. 1), taken to address this key unmet need has been divided into (1) within-hospital steps and (2) Community outreach.

### 4.1 Within-trust action

We employed a multi-level engagement in order to reach all levels of the hospital staff and as a result, patients across all specialties, to ensure that all patients at KCH were aware of the currently offered portfolio of studies. More importantly, we ensured that all the staff at KCH were not only aware of the research portfolio but were actively engaged in promoting research. This was facilitated by engaging senior management to ward staff. We felt it was especially important to engage leaders of EM background to increase relatability among both EM staff and patients.

### 4.2 Community outreach

It was felt that engaging local communities in the dialogue is key to widening the public involvement, participation and understanding of research, outside of a hospital setting. We therefore formed a close collaboration with the local church leaders and community champions who continue to support the initiative.

## 5 Future directions

This initiative, whilst successful, highlighted a fundamental unmet need the resourcing when addressing research recruitment imbalance among the ethnic minority populations. Healthcare centres are in dire need of providing more resources to enable promotion and engagement of ethnic minority populations in research and more widely, decision-making regarding health management. It is therefore imperative that the budget being used to utilise such models as the King's Model is carved out, given that as demonstrated by our initiative, it leads to a significant improvement in addressing the gap highlighted by numerous publications, including Lau et al. [4]. The King's Model, when resources are available, proposes a real-world working approach when utilised, though its feasibility ought to be tested on a wider scale.

Considering the proposed Model, we recognise that there are several factors which may influence its implementation and effectiveness. We therefore propose the MAADE scheme which outlines key factors for consideration in long term utilisation of the King's Model.

It is too early to see if the King's Model is replicable nationally or outside of the NHS, however further research should focus on the implementation of the King's Model and its efficacy on a wider scale. Reports regarding disparities in representation of ethnic minority populations in research have been highlighted over the past decades and have become more paramount following the Covid-19 pandemic. The King's Model provides a world-first novel approach in addressing this gap and supports the need for further work to ensure ethnic minority populations are represented in research at all levels, to ensure participation in research is truly universal for all.

## Ethics approval and consent to participate

As this was an implementation of a new strategy to increase recruitment, no ethnical approvals were sought. Consent for publication All authors reviewed the manuscript and consented for publication.

## Availability of data and materials materials-methods

The datasets analysed during the current study are available from the corresponding author on reasonable request.

## Authors' contributions

Conceptualisation: AP, KRC, JEO; Data analysis: AP, MQ, AM; Supervision: KRC, CG, AG, JEO; Writing – Original draft: AP; Writing – Review and Editing: AP, KRC, YHL, AM, AQ, SOD, KL, MA, AG, SB, AL, OA, AMM, JEO. All authors read and approved the final manuscript.

## Funding

Dr Josephine Ocloo, King's College London is supported by the National Institute for Health Research (NIHR) Applied Research Collaboration South London (NIHR ARC South London) at King's College Hospital NHS Foundation Trust. The views expressed are those of the author[s] and not necessarily those of the NIHR or the Department of Health and Social Care.

## Declaration of competing interest

Authors declare no conflicts of interest.

## List of abbreviations

**COVID-CNS**: COVID-19 Clinical Neuroscience Study
**EM**: ethnic minorities
**NOVAVAX**: A Phase 3, Randomised, Observer-Blinded, Placebo-Controlled Trial to Evaluate the Efficacy and Safety of a SARS-CoV-2 Recombinant Spike Protein Nanoparticle Vaccine (SARS-CoV-2 rS) with Matrix-M1™ Adjuvant in Adult Participants 18–84 Years of Age in the United Kingdom
**PD**: Parkinson's disease
**PHOSP-COVID**: Post-hospitalisation COVID-19 study
