# Supplementary material for: Addressing the gap for racially diverse research involvement: The King's Model for minority ethnic research participant recruitment
Source: Public Health Pract (Oxf). 2023 Sep 10;6:100426. doi: 10.1016/j.puhip.2023.100426 (PMC10511795; doi:10.1016/j.puhip.2023.100426)
Supplement: Multimedia component 1 [file mmc1.docx]

Dear Editor,

London, United Kingdom

30 July 2023

We thank you very much for your insightful comments. Please find below our responses along with the revised version of the manuscript (file name: KingsModel_KRC_AP_2023_revised_2_TC.docx) along with the clean version.

We are looking forward to you reading our amended work and we do indeed hope you will find it agreeable.

Should you have any further comments, or should you feel that additional revisions are required, please do not hesitate to contact us.

With kind regards and best wishes,

Prof K Ray Chaudhuri and A Podlewska

*Please revise articulating clearly what the 'King's Model' is. This is unclear in the piece. the model needs to be introduced clearly in the introduction and then illustrated in the method - what is it? what did you do with it, when and to whom. Please make this explicit. For example - in the abstract 'the king's model' appears in the results with nothing in the preceding text as to what that model is? PHIP likes to share practice - wat model are you trying to share? How was this model devised? How do you propose others should use it? What other models are in place? etc etc Simplify and clarify. It is really unclear what the figures add? Can this be explained better please, use headings and notes under figures as need be.*

*We thank you for your insightful review. We have clarified parts of introduction, methods and discussion to suit the requirements better. All amended and added clarifications are outlined in yellow in the Track Changes version of the manuscript. We hope that the added explanations provide a better understanding of the proposed Model, which aims to propose a set of steps and actions a research centre can take to promote diverse recruitment into clinical trials. We aim to use the current work as a foundation for future research to validate the proposed steps as a scheme of work to effectively increase diversity in clinical trial participation.*

*Last para of intro - remove word 'glaring' - remain objective in writing. Thank you, this has been removed.*

*Also, please remove references from the abstract - reference the main article only. Thank you, this has been removed.*

*Please highlight the revisions in text with any resubmission - the changes must be easy to see.* Please find attached a version with Track Changes, which have also been highlighted in yellow.
